# Intraspecific variation in a predator changes intertidal community through effects on a foundation species

**DOI:** 10.1002/ece3.10131

**Published:** 2023-06-06

**Authors:** Gina M. Contolini, Eric P. Palkovacs

**Affiliations:** ^1^ Department of Ecology and Evolutionary Biology University of California Santa Cruz Santa Cruz California USA

**Keywords:** community diversity, eco‐evolutionary dynamics, intertidal, niche construction, predation

## Abstract

Intraspecific variation is an important form of biodiversity that can alter community and ecosystem properties. Recent work demonstrates the community effects of intraspecific variation in predators via altering prey communities and in foundation species via shaping habitat attributes. However, tests of the community effects of intraspecific trait variation in predators acting on foundation species are lacking despite the fact that consumption of foundation species can have strong community effects by shaping habitat structure. Here, we tested the hypothesis that intraspecific foraging differences among populations of mussel‐drilling dogwhelk predators (*Nucella*) differentially alter intertidal communities through effects on foundational mussels. We conducted a 9‐month field experiment where we exposed intertidal mussel bed communities to predation from three *Nucella* populations that exhibit differences in size‐selectivity and consumption time for mussel prey. At the end of the experiment, we measured mussel bed structure, species diversity, and community composition. While exposure to *Nucella* originating from different populations did not significantly alter overall community diversity, we found that differences in *Nucella* mussel selectivity significantly altered foundational mussel bed structure, which in turn altered the biomass of shore crabs and periwinkle snails. Our study extends the emerging paradigm of the ecological importance of intraspecific variation to include the effects of intraspecific variation on predators of foundation species.

## Graphical abstract

Recent work demonstrates the community effects of intraspecific variation in predators via altering prey communities and in foundation species via shaping habitat attributes, but tests of the community effects of intraspecific trait variation in predators acting on foundation species are lacking. We used a 9‐month field experiment to test the hypothesis that intraspecific foraging differences among populations of mussel‐drilling dogwhelk predators differentially alter intertidal communities through effects on foundational mussels. We found that differences in dogwhelk prey selectivity significantly altered foundational mussel bed structure, which in turn altered the biomass of shore crabs and periwinkle snails, extending the emerging paradigm of the ecological importance of intraspecific variation to include the effects of intraspecific variation in predators of foundation species.

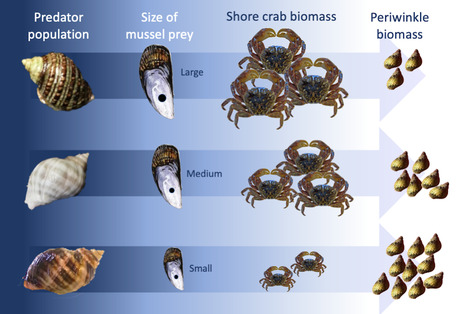

## 1 INTRODUCTION

Intraspecific trait variation can have important ecological effects, altering interactions and reshaping communities and ecosystems (Albert, 2015; Bolnick et al., 2011; Des Roches et al., 2018; Hairston et al., 2005; Harmon et al., 2009; Hughes et al., 2008; Palkovacs et al., 2012; Raffard et al., 2019). Much evidence for the community effects of intraspecific trait variation focuses on predators directly altering prey communities (Butler, 1989; Des Roches et al., 2013; Hughes et al., 2015; Katano, 2011; Matthews et al., 2016; Palkovacs & Post, 2009; Stemp et al., 2020; Wood et al., 2019) and genetic variation in foundation species (Bangert et al., 2008; Hays et al., 2021; Hughes et al., 2008; Whitham et al., 1999). However, predators of foundation species can have indirect effects on communities by altering habitat structure, both through nonconsumptive (Catalán et al., 2021) and consumptive effects (Hughes et al., 2015; Navarrete, 1996; Paine, 1966). Yet the community effects of trait variation in predators of foundation species remain untested.

Rocky intertidal communities are often shaped by foundational mussels. In the California Current Ecosystem, the abundance and size structure of intertidal California mussels (*Mytilus californianus*) shape the community of coexisting species. The introduction of predators on mussels, either sea stars (*Pisaster*) or dogwhelks (*Nucella*), reduces the abundance of mussels, not only creating opportunities for competing species but also decreasing habitat for colonization and refuge for commensal species like small gastropods, worms, and crustaceans (Lafferty & Suchanek, 2016; Menge et al., 2021; Navarrete, 1996). While prior work has focused on the community effects of mussel predator presence or absence (Menge et al., 1994; Paine, 1966), there is a lack of understanding about the community effects of mussel predator intraspecific trait variation.

**FIGURE 1:**
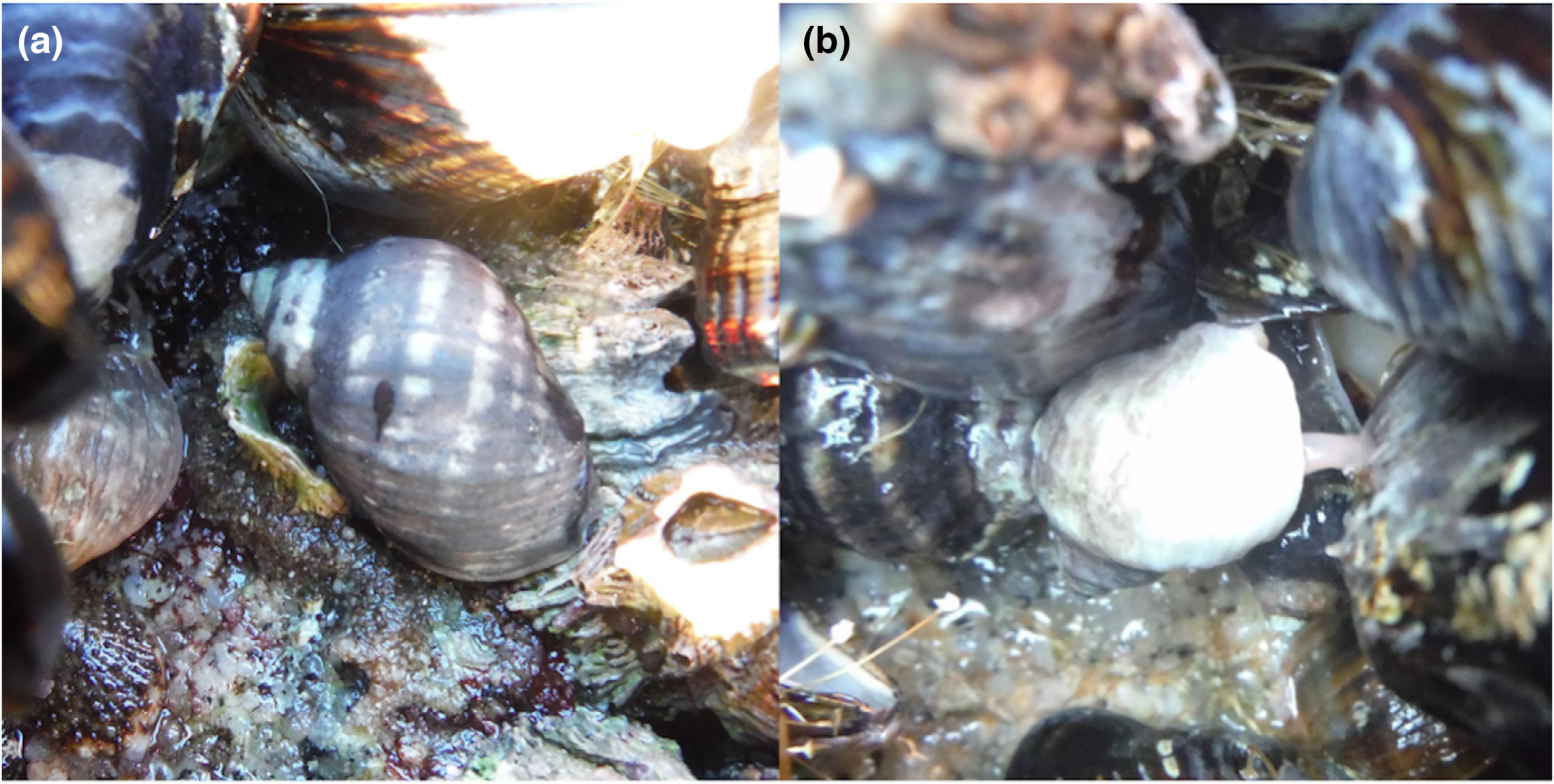
*Nucella* dogwhelk (a) resting and (b) feeding with proboscis inserted into a California mussel.

*Nucella ostrina* and *Nucella emarginata* are sister species of intertidal dogwhelks commonly found on the west coast of North America (Figure 1). These species live in mid‐intertidal California mussel beds and feed primarily on mussels and barnacles by drilling (Clelland & Saleuddin, 2000; West, 1986). In central California, their distributions overlap, and the two species are largely indistinguishable genetically, morphologically, and ecologically (Marko, 1998, 2005, Marko et al., 2014, Raimondi pers. comm.), including in a recent neutral genetic analysis of the populations used in the present study (Contolini, Reid, & Palkovacs, 2020). Therefore, we consider trait differences among these three populations equivalent to intraspecific trait variation and hereafter refer to the species collectively as “*Nucella*.” *Nucella* have crawl‐away larvae that develop and hatch in the same intertidal area as their parents. This life history limits gene flow and can lead to local adaptation in foraging traits (Dawson et al., 2014; Marko, 1998; Sanford & Worth, 2009). Indeed, *Nucella* exhibits population‐level differences in size selectivity and consumption rate of mussels, which are traits that could alter the size structure of mussel beds (Contolini, Kroeker, & Palkovacs, 2020; Contolini, Reid, & Palkovacs, 2020).

Here we used a 9‐month field experiment to test the prediction that population‐level variation in *Nucella* consumption rate and size selectivity of mussel prey indirectly alters intertidal community composition by changing the physical structure of the mussel bed on which other organisms depend for inhabitable space. We predict that *Nucella* populations will differentially alter mussel density and size, and these changes will cascade to affect species diversity and community composition of organisms living within and atop the mussel bed matrix. Our study tests the role of intraspecific variation in predator foraging in communities through effects on foundation species.

## 2 MATERIALS AND METHODS

### 2.1 Nucella source sites

**FIGURE 2:**
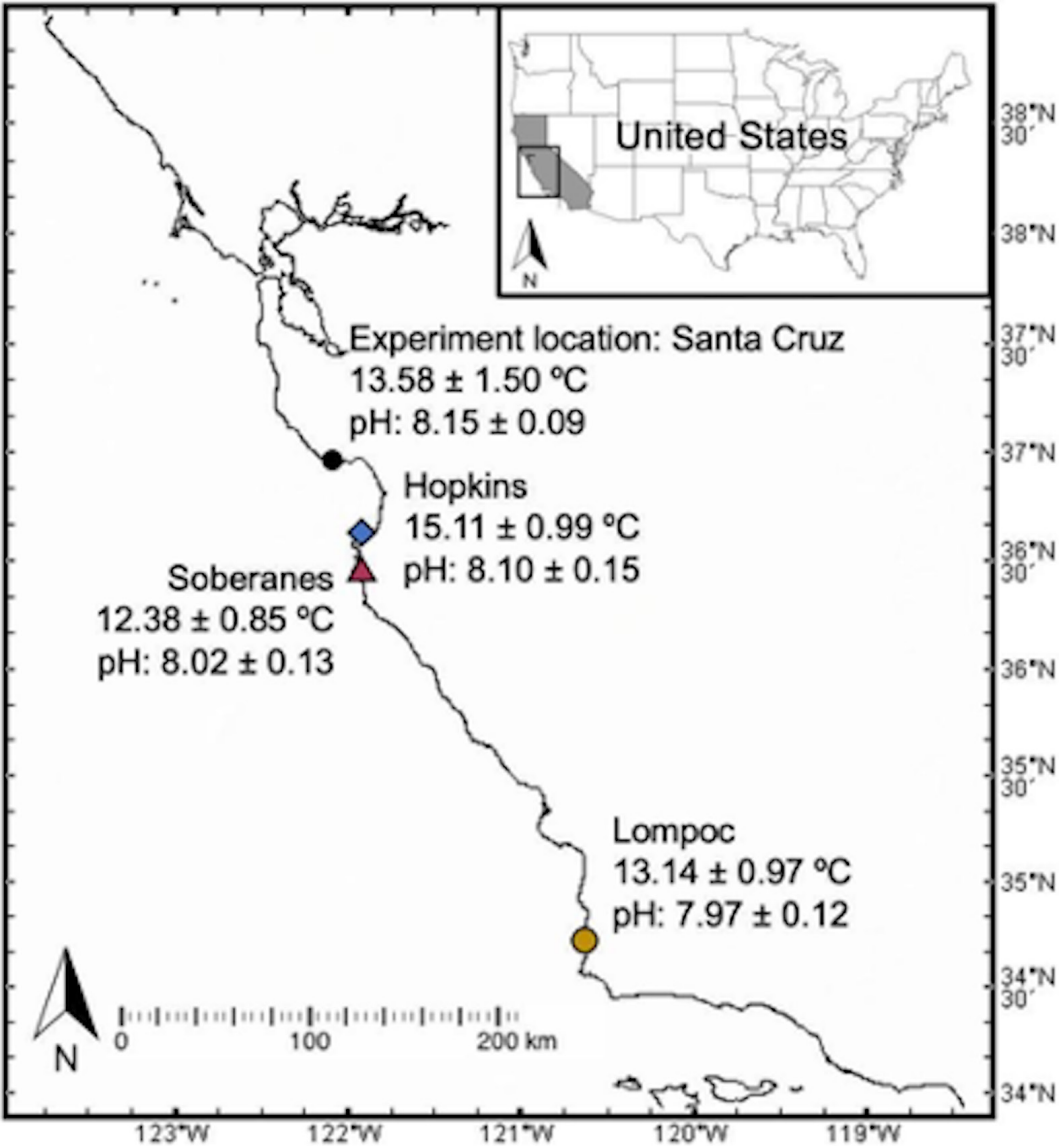
Map of study sites and experiment location with mean ± SD temperature and pH. Santa Cruz temperature is from a mid‐zone intertidal logger for the duration of the experiment (17 Oct 2017 to 1 Aug 2018 [Multi‐Agency Rocky Intertidal Network (MARINe) et al., 2017, 2018]). Hopkins, Soberanes, and Santa Cruz pH data are from intertidal pH sensors from 15 July to 22 September. Lompoc data are from an offshore sensor (Purissima) from the same date range in 2011 (Rivest et al., 2016). More details of the pH and temperature regimes can be found in Table S1 and Chan et al., (2017).

We collected adult *Nucella* from three mid‐intertidal California mussel bed sites in September 2017: Hopkins Marine Reserve (19 Sep; 36.62°, −121.91°; *N* = 99), Soberanes Point (19 Sep; 36.45°, −121.93°; *N* = 60), and Lompoc Landing (21 Sep; 34.72°, −120.61°; *N* = 60; Figure 2). These sites naturally experience different seawater temperature and pH regimes. On average, Hopkins experiences the warmest mean seawater temperature and highest mean pH seawater; Lompoc is intermediate in mean temperature and lowest in mean pH; and Soberanes has the coolest mean seawater temperature with intermediate mean pH (Table S1; Chan et al., 2017). On average, adult *Nucella* shell lengths from these three sites are not significantly different (mean ± SE Hopkins 23.30 ± 0.53 mm, *N* = 35; Soberanes 24.27 ± 0.64 mm, *N* = 46; Lompoc 23.60 ± 0.83 mm, *N* = 38; Contolini, Reid, & Palkovacs, 2020). *Nucella* from Lompoc on average drill larger mussels (mean ± SE 54.18 ± 8.73 mm mussel length compared with 41.88 ± 1.35 and 42.13 ± 2.87 mm for Hopkins and Soberanes, respectively) and can consume a California mussel several days faster than *Nucella* from both Hopkins and Soberanes (mean ± SE 25.52 ± 2.92 d compared with 31.10 ± 2.68 and 27.52 ± 3.27 d; Contolini, Reid, & Palkovacs, 2020, Contolini, Kroeker, & Palkovacs, 2020). *Nucella* were held in filtered, flowing seawater in the University of California, Santa Cruz Long Marine Lab in Santa Cruz, CA until the start of the experiment on 17 October 2017. Scientific collecting was performed under permits from California State Parks, California Dept. of Fish and Wildlife (SCP #13169), Vandenberg Air Force Base, and Hopkins Marine Station. Intertidal hardware installation and *Nucella* transplantation were performed under a National Oceanic and Atmospheric Administration Office of National Marine Sanctuaries permit (MBNMS‐2017‐025) and California Dept. of Fish and Wildlife and Transplantation Authorization #170913.

### 2.2 Mussel bed predation experiment

To test the effects of population‐level variation in *Nucella* consumption rate and size selectivity of mussels, we performed a common garden experiment at Terrace Point in Santa Cruz, CA, USA (36.94°, −122.06°; Figure 2). Terrace Point is an exposed, gently sloping, south‐facing site with a substrate of consolidated mudstone and multiple rocky benches separated by narrow sandy beaches. In the mid zone, the California mussels are the dominant primary space‐holding species, forming a monoculture covering tens of square meters in a single layer on the horizontal surfaces. The mussel bed creates habitat for mobile animals such as gastropods, crabs, and worms; sedentary and sessile animals such as encrusting worms and barnacles; and algae including scouring pad algae (*Endocladia muricata*), Turkish towel (*Mastocarpus* spp.), and red turf algae (*Calacanthus* sp.). Terrace Point on average has seawater temperatures greater than that of Soberanes and Lompoc and lower than Hopkins, and of the four sites, it has a higher mean and lower standard deviation pH (Table S1).

In the experiment, we outplanted *Nucella* from all three populations to an experimental array of cages and let them feed within their assigned cages for 9 months. The cage array had a total area of 3.4 m^2^ on an existing bench of continuous, level California mussel bed in the mid zone (Figure S1). Mean *Nucella* shell length per cage initially was 24.22 ± 0.64 mm (mean ± SD; *N* = 24) and did not differ significantly among populations (ANOVA *F*_2,21_ = 0.006, *p* = .99; Figure S2a). Mean *Nucella* mass per cage initially was 2.34 ± 0.24 g and also did not differ significantly among populations (Kruskal–Wallis test, X^2^_21_ = 23, *p* = .3; Table S2; Figure S2b). Any *Nucella* that died or were lost were replaced with one of a similar size from the same source population held in tanks in the nearby marine lab. We created the array by clearing all biological material 10 cm around thirty‐two 20 × 20 cm natural mussel bed plots such that each was surrounded by a border of bare rock. We used existing mussel bed so we could test the responses of fully established mussel bed communities; for this reason, it was not possible to measure community diversity and composition within each cage at the start of the experiment to obtain a baseline because doing so required destructive sampling. We installed cages made from 0.4 cm Vexar mesh and secured them to the rock using stainless steel lag screws, washers, and marine epoxy (Z‐Spar splash zone compound). Cages were 20 × 20 × 8 cm with removable lids secured with cable ties. We arranged the cages in rows parallel to the shore and assigned them to one of four treatments: five adult *Nucella* from one of the three populations or no *Nucella* (control), each replicated eight times and in a randomized block design (Figure S3). Blocks (*N* = 8) ensured treatments were approximately equally exposed to edge and tidal effects and allowed us to account for this natural variation in statistical models. *Nucella* density was within the range of densities observed at the source sites (<1 to 10 m^−2^; Multi‐Agency Rocky Intertidal Network (MARINe), 2021, pers. obs.). We marked *Nucella* with bright nail polish and uniquely numbered all individuals with bee tags (Bee Works, Canada) attached to the shell with cyanoacrylate glue. To control for differences in *Nucella* size, we measured *Nucella* starting and ending shell length from the shell apex to the tip of the anterior canal using digital calipers. We measured *Nucella* starting and ending mass by drying snails thoroughly with paper towels, squeezing excess water out from behind the operculum, and using an analytical scale (Mettler Toledo AG104). We initiated the experiment on 17 October 2017 by adding tagged and premeasured *Nucella* to assigned cages. We opened all cages biweekly to photograph the surface (Canon PowerShot D30), record drilled mussels, collect dislodged mussels, remove invading non‐experimental *Nucella*, and replace dead or lost *Nucella* with one of a similar size from the same population (23 from Soberanes, 5 each from Hopkins and Lompoc). We monitored temperature (water and air) at the site and inside two cages every 15 min using HOBO temperature loggers (Onset Computer Corporation; Table S3) and compared this with previously published temperature regimes at each of the *Nucella* population sites (Menge et al., 2015; Multi‐Agency Rocky Intertidal Network [MARINe] et al., 2017, 2018; Rivest et al., 2016) (Table S1). The average mid zone temperature (air and water combined) at the experiment site was 13.31 ± 1.68 SD°C (Table S3). Inside cages, the mean combined air and water temperature was on average 0.52°C higher than the temperature of the mid‐zone mussel bed outside of cages. Nine months later, on 29 July–1 August 2018, we measured the *Nucella* in the cages and removed and froze all mussels and their associated communities, including all organisms within and on top of the mussel bed matrix, at −20°C for further analyses.

### 2.3 Mussel bed structure and community composition

We characterized the direct effects of *Nucella* on the mussel beds at the end of the experiment by measuring the sizes of drilled and remaining mussels using digital calipers. To characterize the ecological community within the mussel bed matrix, we identified all organisms within the mussel beds to the lowest possible taxon (https://seanet.stanford.edu/; Light & Smith, 2007). We sorted all organisms, cleaned them of sand and large debris by hand or by rinsing them with fresh water, dried them in a 56°C oven (Chicago Surgical & Electrical Co. Imperial II, Thelco Precision Model 2, or Quincy Lab Model 40GC) for 7 d, and measured the dry mass of each taxon per cage using an analytical scale (Mettler Toledo AG104). Mass of all shelled organisms included shells. As we were unable to obtain accurate measurements of algal biomass due to it fracturing into small pieces when handled, we instead calculated the final percent cover of algae on top of the mussel beds, where most algae were located, using the bed surface photographs and ImageJ image analysis software v. 1.51 s (Abràmoff et al., 2004).

### 2.4 Statistical analyses

We used analysis of variance (ANOVA) or, when data transformation did not normalize non‐normal error distributions, Kruskal–Wallis tests to test for significant differences in *Nucella* sizes, number of drilled and remaining mussels, mean size of drilled and remaining mussels, community diversity (Shannon–Wiener diversity), and biomass of individual taxa among *Nucella* treatments. We used ANOVA models with type II sums of squares for number and size of drilled and remaining mussels, with main effects *Nucella* population and block to account for variation among blocks. We transformed variables when necessary to meet assumptions of normality and homogeneity of variances. We also performed permutational multivariate analysis of variance (PERMANOVA) to test for significant effects of *Nucella* population on community composition using Bray–Curtis dissimilarity community matrices, accounting for block as a main effect, and using 999 permutations. We used similarity percentages (SIMPER) with Bray–Curtis dissimilarities to find which species most discriminated between treatment communities (package *vegan*; Oksanen et al., 2018).

We tested for indirect effects of *Nucella* on diversity through changes in the mussel bed structure first using ANOVA to test for significant differences in the number and size of drilled and remaining mussels among treatments. We then used linear regressions to test for significant relationships between the number and size of drilled or remaining mussels and Shannon‐Wiener diversity or biomass of influential taxa, as identified using SIMPER. Finally, we used piecewise structural equation modeling (SEM) to examine direct and indirect mechanisms by which population‐specific patterns of *Nucella* foraging could influence the ecological community (package *piecewiseSEM* v. 2.1.0; Lefcheck, 2016). Piecewise SEM is a type of confirmatory path analysis that joins suites of interrelated variables in a network and can incorporate complex model structures such as categorical exogenous variables with many factor levels—in our case, *Nucella* population had three levels. The model consisted of analysis of variance and linear models using *Nucella* population as the exogenous variable and drilled mussel size and biomass of individual taxa as endogenous variables. All analyses were performed in the *R* statistical environment v. 4.1.3 (R Foundation for Statistical Computing, 2022) using *RStudio* v. 2022.02.0 (RStudio Team, 2022).

## 3 RESULTS

### 3.1 Nucella growth

By the end of the experiment, *Nucella* from Lompoc and Soberanes on average increased in length (by 1.41 and 1.76 mm, respectively), but Hopkins *Nucella* did not, resulting in significant differences in final mean shell length among populations (ANOVA, block *F*_7,14_ = 0.72, *p* = .66; population *F*_2,14_ = 8.58, *p* = .004; Tukey HSD pairwise comparisons with Hopkins, *p* < .01; Soberanes‐Lompoc, *p* = .99; Table S4; Figure S2a). Similarly, *Nucella* from Lompoc and Soberanes on average increased mass, resulting in significant differences in mean mass between all populations by the end of the experiment (ANOVA, block *F*_7,14_ = 0.77, *p* = .62, population *F*_2,14_ = 15.31, *p* < .001; Tukey HSD *p* ≤ .05 for all pairwise comparisons; Table S4; Figure S2b).

### 3.2 Direct effects of *Nucella* on mussel bed structure

**FIGURE 3:**
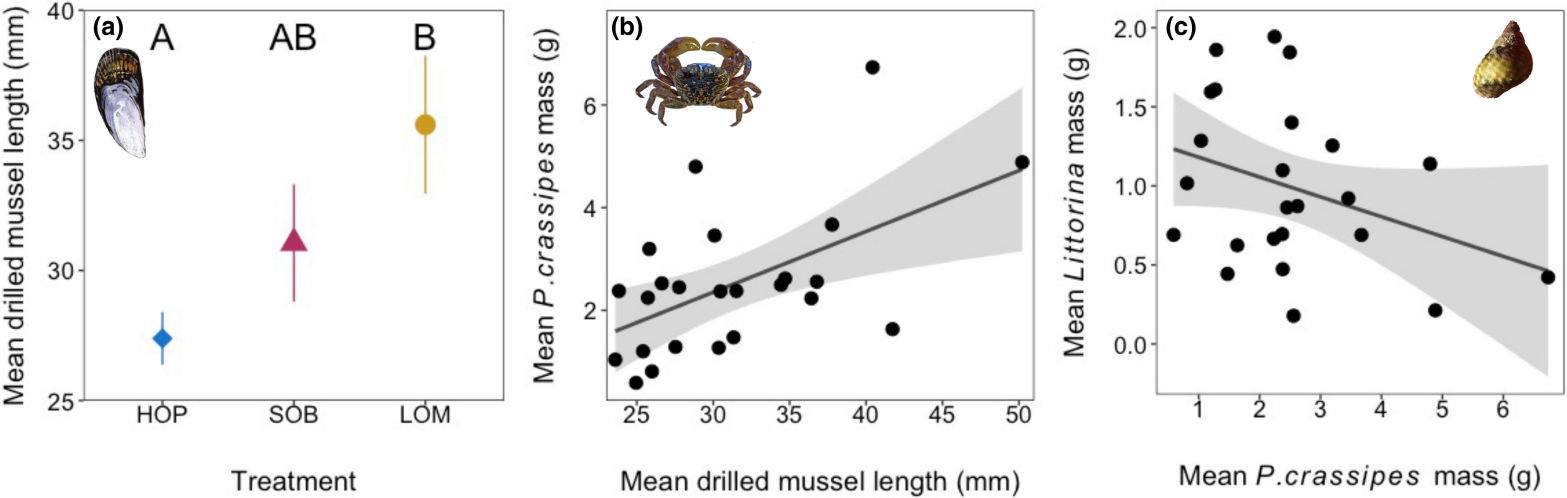
Effects of *Nucella* population on mussel bed structure and species biomass. (a) Mean ± SE drilled mussel length by *Nucella* population (*N* = 24). (b) Linear regression ±95% CI of mean *P. crassipes* biomass versus mean drilled mussel length (*N* = 24, adjusted *R* ^2^ = .46, *p* = .02). (c) Linear regression ±95% CI of mean *Littorina* biomass versus mean *P. crassipes* biomass (*N* = 24, adjusted *R* ^2^ = .45, *p* = .02).

Over the course of the experiment, *Nucella* from each population drilled over 150 mussels (*N* = 622 total) and visibly deteriorated mussel beds (Table S5). There were no significant differences in the number of mussels drilled among *Nucella* populations (ANOVA, block *F*_7,14_ = 0.50, *p* = .82; population *F*_2,14_ = 1.05, *p* = .30). The control cages had a low number of drilled mussels (*N* = 30) from small, transient native dogwhelk invaders that were immediately removed once found (mean ± SD 7.00 ± 4.10 invaders per 2‐week sample period, and 133 total over the course of 9 months). The control treatment had significantly fewer drilled mussels than *Nucella* treatments (ANOVA, block *F*_7,21_ = 0.47, *p* = .84; treatment *F*_3,21_ = 14.63, *p* < .001; Tukey HSD *p* < .01 for populations paired with control). There were significant differences in the mean size of drilled mussels among *Nucella* populations (Table 1). The Lompoc population drilled largest mussels (mean ± SD 35.6 ± 7.49 mm), significantly larger than Hopkins (27.4 ± 2.86 mm; Tukey HSD *p* = .04), but not Soberanes (31.10 ± 6.38 mm; Tukey HSD *p* = .45; Figure 3a). The mean drilled mussel length in the control treatment was significantly smaller than in *Nucella* treatments (mean ± SD 15.9 ± 6.48 mm; ANOVA, block *F*_7,21_ = 1.15, *p* = .37; treatment *F*_3,21_ = 16.07, *p* < .001; Tukey HSD *p* < .01 for all populations paired with control). There were no significant differences in log mean number or mean length of remaining mussels among *Nucella* populations (ANOVA for log mean number of remaining mussels: block *F*_7,14_ = 0.56, *p* = .77; population *F*_2,14_ = 0.97, *p* = .40; ANOVA for mean length of remaining mussels: block *F*_7,14_ = 0.93, *p* = .52, population *F*_2,14_ = 0.62, *p* = .55; Table S6).

**TABLE 1:** Summary of ANOVA model of mean drilled mussel length versus *Nucella* population.

	Sum sq	DF	*F*	*p*
Block	251.84	7	1.04	.45
*Nucella* population	270.97	2	3.92	.04*
Residuals	483.44	14		

*Note:* Significant values (*p* ≤ .05) are indicated with an asterisk (*).

### 3.3 Direct effects of *Nucella* on community composition

The source population of *Nucella* did not significantly affect Shannon‐Wiener diversity (ANOVA, block *F*_7,14_ = 2.21, *p* = .10; population *F*_2,14_ = 0.02, *p* = .98; Table S7) or species composition (PERMANOVA, block *F*_7,14_ = 1.37, *p* = .19; population *F*_2,14_ = 0.78, *p* = .61; Table S8). SIMPER analysis showed *Tegula funebralis*, *Pachygrapsus crassipes*, *Anthopleura* spp., *Lottia* spp., *Nereis* spp., *Littorina* spp., *Septifer bifurcatus* biomass, and algal cover comprised over 90% of cumulative community differences among treatments. Of these taxa, *Nucella* population was significantly related to *P. crassipes* (shore crab) biomass (ANOVA, block *F*_7,14_ = 2.49, *p* = .07; population *F*_2,14_ = 3.86, *p* = .05; Table S9), where crabs had significantly more biomass in treatments with *Nucella* from Lompoc (mean biomass ± SD 3.37 ± 1.71 g) compared with Soberanes (1.91 ± 1.04 g; Tukey HSD *p* = .05), but not Hopkins (2.25 ± 1.17 g; Tukey HSD *p* > .1).

### 3.4 Indirect effects of *Nucella* on community composition

**FIGURE 4:**
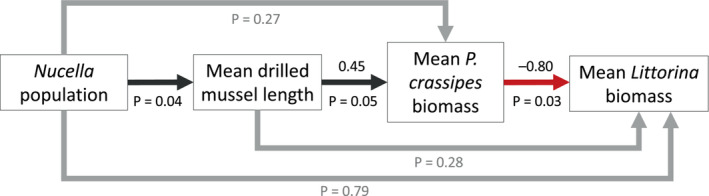
Path diagram showing results of the structural equation model for *Nucella* population, mean drilled mussel length, mean *Pachygrapsus crassipes* biomass, and mean *Littorina* spp. biomass in the mussel bed. Standardized regression coefficients are shown above paths and significances are shown below. Red arrows indicate negative relationships. Nonsignificant paths are gray.

**FIGURE 5:**
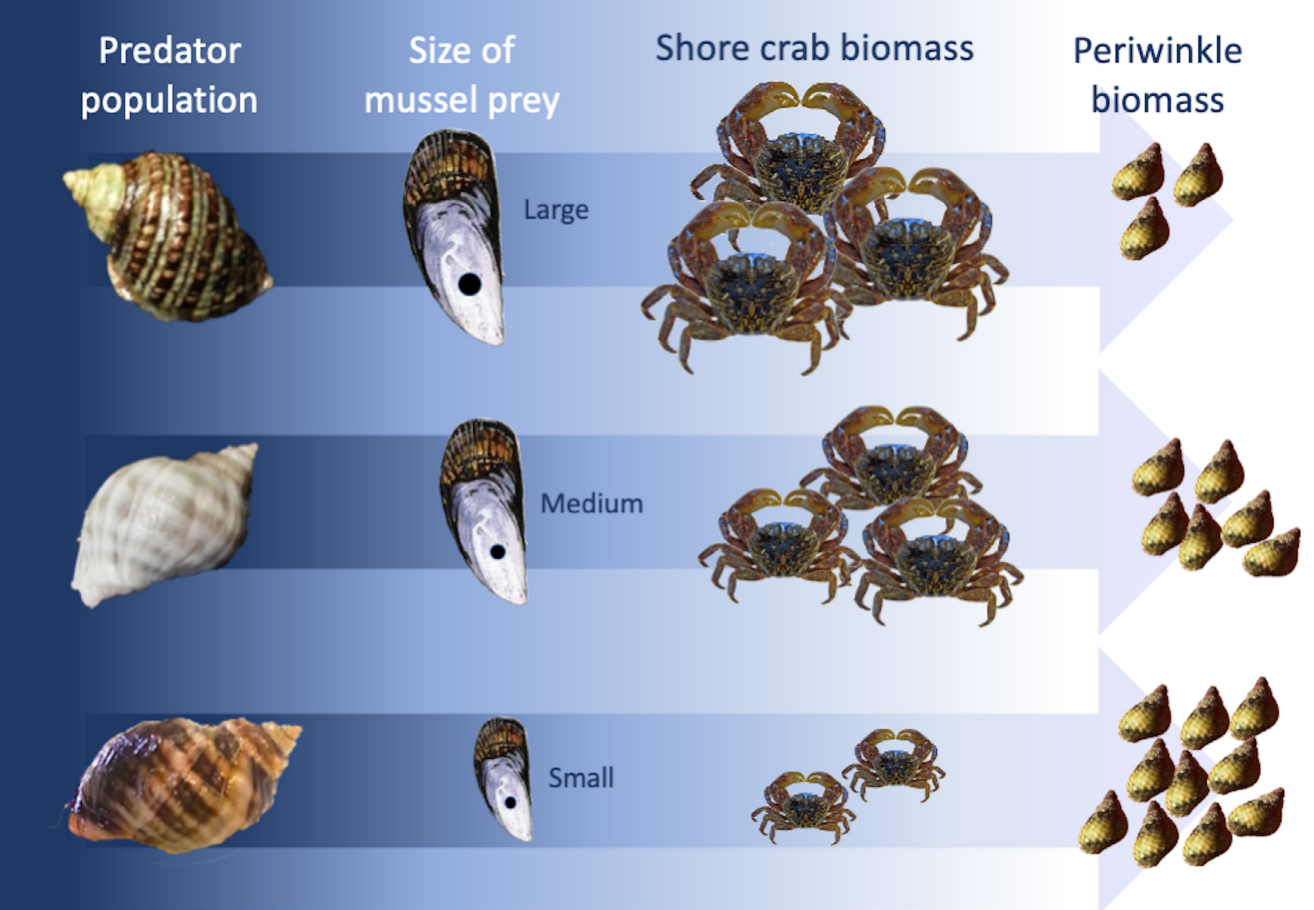
Schematic showing effects of intraspecific variation in *Nucella* predation on *Pachygrapsus crassipes* (shore crabs) and *Littorina* spp. (periwinkle snails). Size selectivity of mussel prey differed for each *Nucella* population. This differential consumption led to differences in shore crab biomass, in turn altering periwinkle snail biomass.

Linear models showed mean drilled mussel length was significantly related to *P. crassipes* biomass (Table 2; Figure 3b), *Lottia* spp. biomass (Table S10), and *Littorina* spp. (Table S11). Mean drilled mussel length was not significantly related to other taxa or Shannon‐Wiener diversity. *P. crassipes* biomass was significantly negatively related to *Littorina* spp. biomass (Table 3; Figure 3c). Considered together in the structural equation model, there were significant path coefficients from *Nucella* population to mean drilled mussel length (*p* = .04, *R* ^2^ = .52), mean drilled mussel length to mean *P. crassipes* biomass (coefficient = 0.45, *p* = .05, *R* ^2^ = .74), and mean *P. crassipes* biomass to mean *Littorina* spp. biomass (coefficient = −0.80, *p* = .03, *R* ^2^ = .68), but not from *Nucella* population to mean *P. crassipes* biomass (*p* = .15), *Nucella* population to mean *Littorina* biomass (*p* = .71), or mean drilled mussel length to mean *Littorina* biomass (*p* = .28; Table S12; Figures 4 and 5).

**TABLE 2:** ANOVA table for linear regression of mean *Pachygrapsus crassipes* biomass versus mean drilled mussel length.

	Sum sq	DF	*F*	*p*
Block	16.45	7	2.14	.10
Mean drilled mussel length	9.64	1	8.79	.01*
Residuals	16.44	15		

*Note*: Residual standard error: 1.047 on 15 degrees of freedom. Multiple *R* ^2^: .650, Adjusted *R* ^2^: .464. *F*‐statistic: 3.485 on 8 and 15 DF, *p*‐value: .018.

Significant values (*p* ≤ .05) are indicated with an asterisk (*).

**TABLE 3:** ANOVA table for linear regression of mean *Littorina* spp. biomass versus mean *Pachygrapsus crassipes* biomass.

	Sum sq	DF	*F*	*p*
Block	3.22	7	3.09	.03*
Mean *P. crassipes* biomass	2.66	1	17.83	.0007*
Residuals	2.24	15		

*Note*: Residual standard error: 0.386 on 15 degrees of freedom. Multiple *R* ^2^: .639, Adjusted *R* ^2^: .447. *F*‐statistic: 3.322 on 8 and 15 DF, *p*‐value: .022.

Significant values (*p* ≤ .05) are indicated with an asterisk (*).

## 4 DISCUSSION

The community effects of intraspecific trait variation are well known for predators shaping prey communities and for bottom‐up effects of foundation species, but not for predators of foundation species. In this study, we tested the effects of mussel predator trait variation on the mussel bed community. We used a 9‐month field experiment to test the hypothesis that population‐level variation in *Nucella* consumption rate and size selectivity of mussel prey indirectly alters intertidal community composition by changing the physical structure of the mussel bed on which other organisms depend. Our results supported this hypothesis. First, our results showed *Nucella* predation significantly affected mussel bed structure, as treatments with *Nucella* had significantly more and larger drilled mussels than the control treatment. Next, by drilling different sizes of mussels, *Nucella* populations differentially altered the structure of the mussel bed and the biomass of certain species living in the mussel bed matrix. These results provide the first evidence of the ecological effects of intraspecific variation in predators of foundation species.

Foraging adaptations could explain the variation in mean drilled mussel size among *Nucella* source populations we observed in our experiment. The process of attacking and consuming mussels is time‐intensive and can take well over a week, during which time *Nucella* is exposed to abiotic and biotic stressors which can act as selective forces (Burrows & Hughes, 1989; Contolini, Kroeker, & Palkovacs, 2020). *Nucella* populations (including *N. emarginata*, *N. ostrina*, and *Nucella canaliculata*) throughout the California Current System show pronounced differences in foraging behaviors that have been linked with abiotic and biotic drivers (Contolini, Reid, & Palkovacs, 2020; Sanford & Worth, 2009). For example, Soberanes and Lompoc naturally experience cooler temperatures than Hopkins (Figure 2; Chan et al., 2017). Under controlled conditions, *Nucella* from Soberanes and Lompoc consume mussels faster than those from Hopkins (Contolini, Kroeker, & Palkovacs, 2020), which is consistent with a pattern of countergradient variation, where elevated feeding rates maintain growth in cooler conditions (Yamahira & Conover, 2002). The mean temperature in our experiment was 2–3°C warmer than at Soberanes and Lompoc, but less than half a degree warmer than at Hopkins, and counter‐gradient feeding adaptations could explain why the cooler source populations attacked larger, more caloric prey and had increased growth.

Our model showed evidence that *Nucella* affected shore crabs indirectly through habitat modification. *Nucella* population itself may not be the strongest direct driver of shore crab biomass because *Nucella* and shore crabs are not known to have strong direct ecological interactions. Mean drilled mussel length, however, may be a stronger direct driver of shore crab biomass because shore crabs use cracks and crevices for living spaces (Bovbjerg, 1960; Kanter, 1977). In our experiment, cages with larger drilled mussels had larger cracks and crevices and higher shore crab biomass, and we observed crabs inhabiting large empty mussel shells (Figure S4). However, mean shore crab biomass was highest in the control treatment where mean shore crab size was smallest, implying that *Nucella* presence had a negative effect on juvenile shore crabs but a positive effect on larger adult shore crabs that increased with drilled mussel size.

*Littorina* spp. (periwinkle snails) were also indirectly affected by *Nucella* through their interaction with shore crabs. Periwinkle snails are small‐bodied (maximum shell length about 25 mm), and prey for shore crabs, so it follows that their biomass would be less in treatments where shore crab biomass was higher (Boulding et al., 2020). Periwinkle biomass was not simply a product of mussel bed size structure because the relationship between periwinkle biomass and mean drilled mussel length was not significant when shore crabs were also included in the model. This is strong evidence that habitat modification by *Nucella* caused significant changes to the trophic interaction between shore crabs and periwinkles that depended on the population‐specific pattern of *Nucella* predation on mussels.

Other studies on population‐specific consumption patterns of marine consumers also confirm the importance of trait variation but do not often test for broader community effects. The presence or absence of salt marsh predators had differential effects on the diversity and biomass of two plant species by amplifying trait variation in herbivores (Hughes et al., 2015) and differences in *Pisaster* body condition were related to mussel cover (Menge et al., 2021). However, neither of these studies tested for effects on entire naturally occurring communities using the habitat formed by foundation species.

Foundation species create habitat for other species but themselves are subject to the effects of predators, which may vary depending on the traits of the predator population. Thus, intraspecific variation in predators of foundation species may cascade to impact entire communities living in biogenic habitat. Our study showed that intraspecific trait variation in *Nucella* predators of foundational mussels altered the biomasses of certain rocky intertidal species via habitat modification. Our results underscore the complexity of ecological and evolutionary processes shaping communities living in biogenic habitats.

## AUTHOR CONTRIBUTIONS

**Gina M. Contolini:** Conceptualization (equal); data curation (lead); formal analysis (lead); funding acquisition (equal); investigation (equal); methodology (lead); project administration (equal); resources (equal); validation (equal); visualization (lead); writing – original draft (lead); writing – review and editing (equal). **Eric P. Palkovacs:** Conceptualization (equal); formal analysis (supporting); funding acquisition (equal); investigation (equal); methodology (supporting); project administration (equal); resources (equal); supervision (lead); validation (equal); visualization (equal); writing – original draft (supporting); writing – review and editing (equal).

## FUNDING INFORMATION

GMC received support from the Myers Ocean Trust, Friends of Long Marine Lab, GAANN P200A150100–17, and the Future Leaders in Coastal Science Award. EPP received partial support from the NOAA Cooperative Institute for Marine Ecosystems and Climate.

## CONFLICT OF INTEREST STATEMENT

The authors declare no conflicts of interest.

### OPEN RESEARCH BADGES

This article has earned an Open Data badge for making publicly available the digitally‐shareable data necessary to reproduce the reported results. The data is available at [10.7291/D1XM4Q].

## Supporting information


Supporting information S1.

Data S1.

Data S2.

Data S3.

Data S4.

Data S5.

## Data Availability

All datasets and R codes used in this study are publicly available on Dryad under DOI 10.7291/D1XM4Q.
