# Supplementary material for: Intraspecific variation in a predator changes intertidal community through effects on a foundation species
Source: Ecol Evol. 2023 Jun 6;13(6):e10131. doi: 10.1002/ece3.10131 (PMC10244894; doi:10.1002/ece3.10131)
Supplement: Supplementary file 1 — Supporting information S1. [file ECE3-13-e10131-s006.docx]

**Supplementary Material for**

Intraspecific variation in a predator effects intertidal community through effects on a foundation species

Gina M. Contolini and Eric. P. Palkovacs

Corresponding author: Gina Contolini

Email: gina@contolini.com

April 2023

**This PDF file includes:**

Figures S1 to S4

Tables S1 to S12

Supplementary References

**Other supplementary materials for this manuscript include the following which will be made available in the online version of the article or on Dryad and linked here if the article is accepted:**

Datasets 1 to 4

1. Contolini_Palkovacs_community_data.csv
2. Contolini_Palkovacs_drilled_mussel_data.csv
3. Contolini_Palkovacs_remaining_mussel_data.csv
4. Contolini_Palkovacs_dogwhelk_data.csv

R script

Contolini_Palkovacs_EE_2023.R

**Figures**


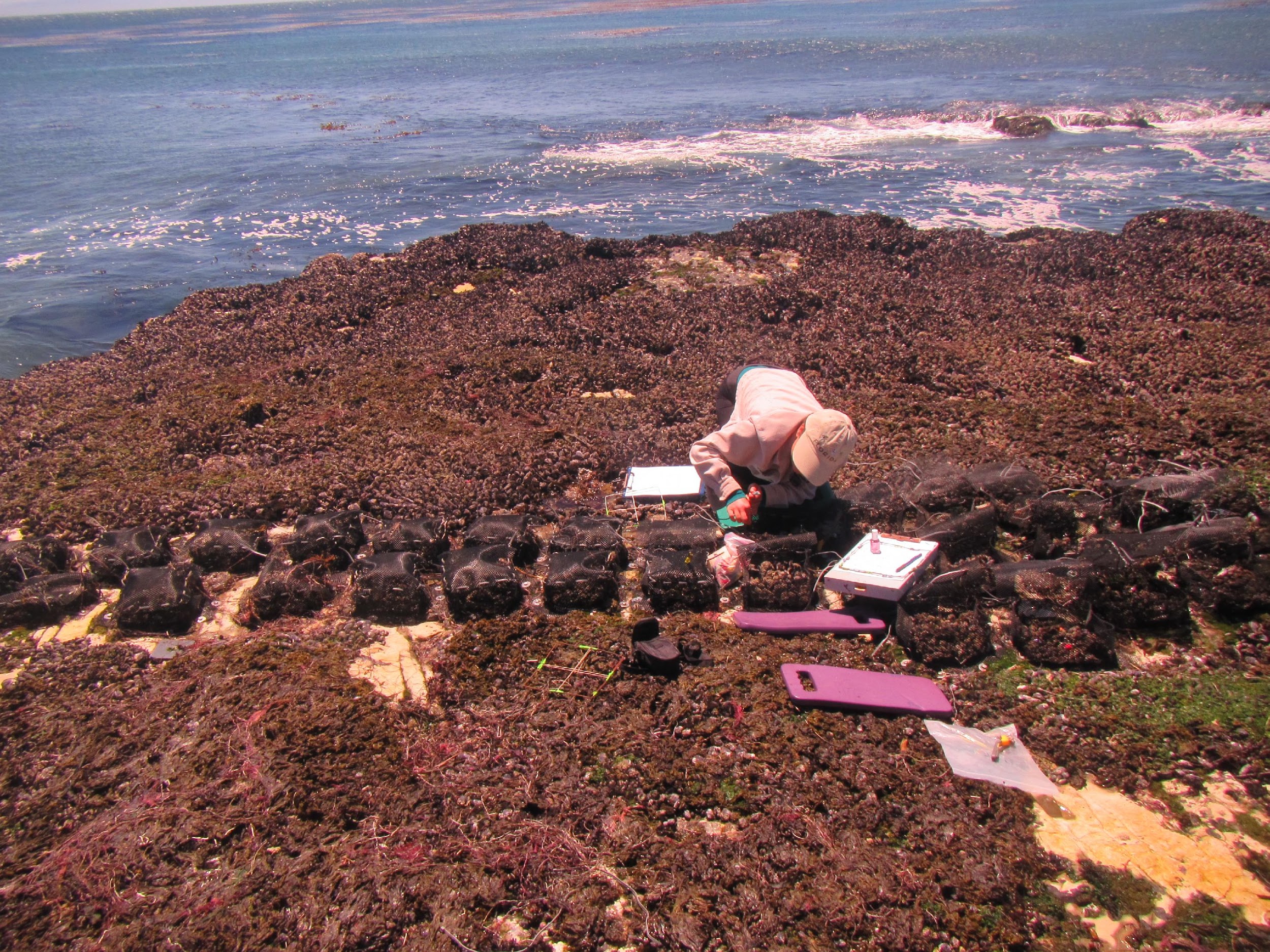


Figure S1. Cage array in the field


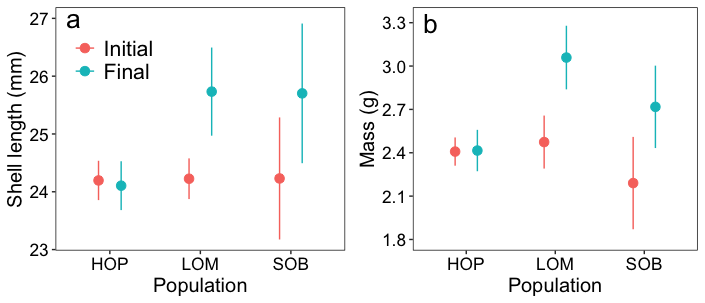


Figure S2. Initial and final *Nucella* (a) shell length and (b) mass (mean ± SD; N = 24).


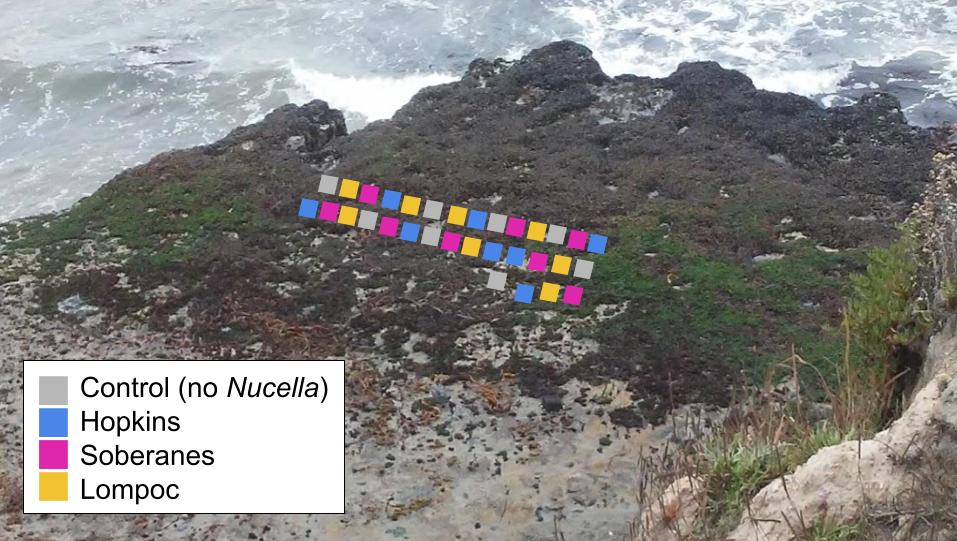


Figure S3. Layout of cage treatments on the intertidal bench. The array was about 4.3 m in length and 3.4 m^2^ in area.


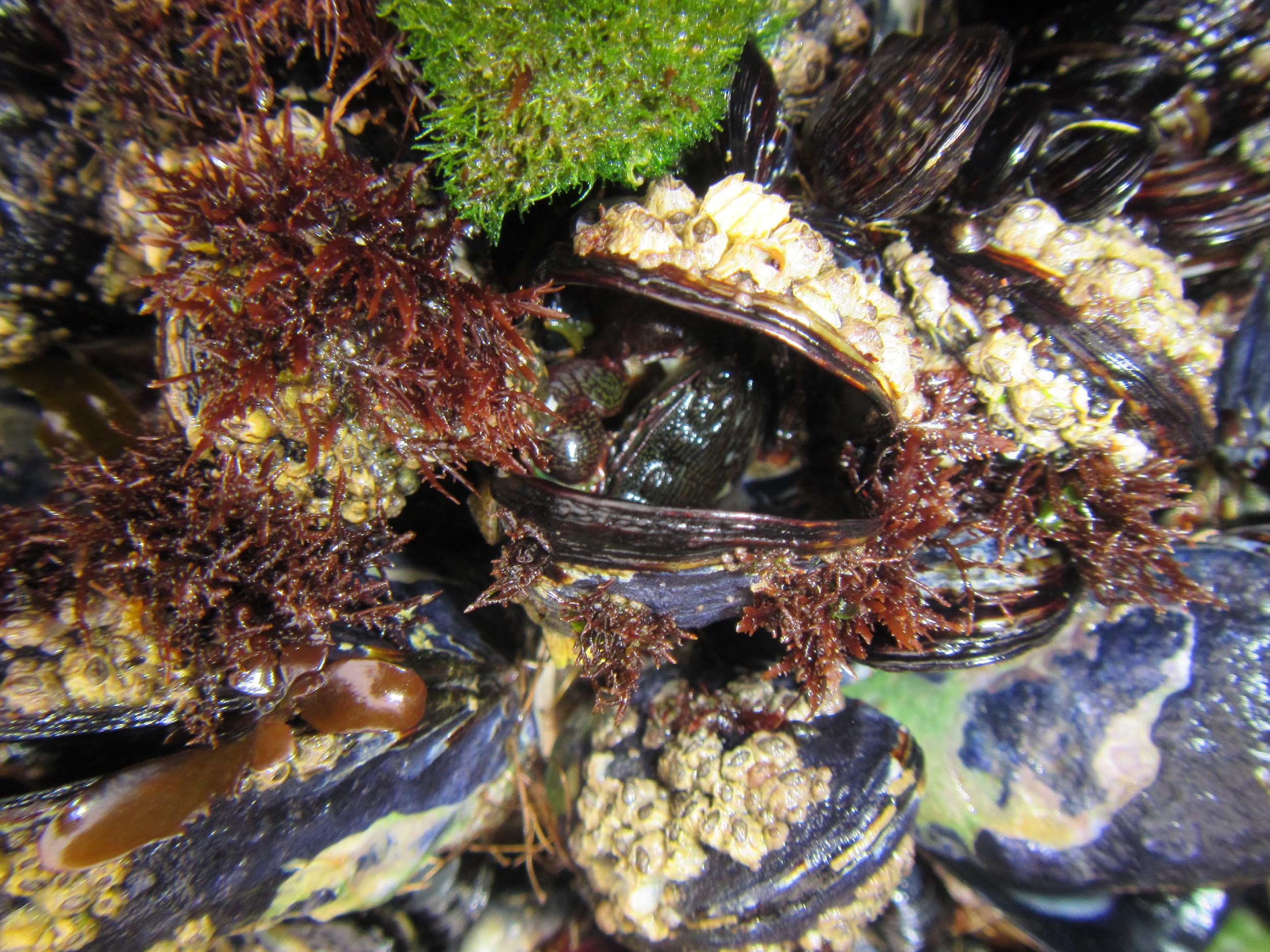


Figure S4. Large *P. crassipes* occupying an empty mussel shell as a result of *Nucella* predation

**Tables**

Table S1. Summary of seawater temperature regime during the experiment (Santa Cruz 2017–18) and during available years and seasons at *Nucella* population origin sites and Santa Cruz.

|  |  | Temperature (°C) | | | | | | pH (total) | | | |
| --- | --- | --- | --- | --- | --- | --- | --- | --- | --- | --- | --- |
|  | | | Mean | Min | Max | SD | N | Mean | Min | Max | SD |
| Santa Cruz 2017–18 | | | 13.58 | 10.35 | 17.51 | 1.50 | 5712 |  |  |  |  |
| Hopkins 2013 | | | 15.11 | 13.00 | 20.27 | 0.99 | 6802 | 8.10 | 7.60 | 8.51 | 0.15 |
| Soberanes 2013 | | | 12.38 | 10.28 | 15.04 | 0.85 | 8103 | 8.02 | 7.50 | 8.29 | 0.13 |
| Lompoc 2011 | | | 13.14 | 11.40 | 15.99 | 0.97 | 4961 | 7.97 | 7.67 | 8.24 | 0.12 |
| Santa Cruz 2012 | | | 14.70 | 12.48 | 17.63 | 0.77 | 13652 | 8.15 | 7.78 | 8.36 | 0.09 |

Santa Cruz 2017–18 temperature is from a mid-zone intertidal logger for the duration of the experiment (17 Oct 2017 to 1 Aug 2018; Multi-Agency Rocky Intertidal Network [MARINe] et al. 2017a, b, 2018). Hopkins 2013, Soberanes 2013, and Santa Cruz 2012 data are from intertidal pH sensors from 15 July to 22 September and Lompoc data are from an offshore sensor (Purissima) from the same day range in 2011 (Rivest et al. 2016). Descriptions of pH regimes at these sites can also be found in Chan et al. (2017).

Table S2. Summary of initial sizes of *Nucella* by population origin

| **Population** | **N** | **Length (mm)** | | | |  | **Mass (g)** | | | |
| --- | --- | --- | --- | --- | --- | --- | --- | --- | --- | --- |
|  |  | **Mean** | **SD** | **Min** | **Max** |  | **Mean** | **SD** | **Min** | **Max** |
| Hopkins | 8 | 24.2 | 0.34 | 23.71 | 24.61 |  | 2.41 | 0.98 | 2.28 | 2.53 |
| Lompoc | 8 | 24.2 | 0.35 | 23.74 | 24.77 |  | 2.47 | 0.18 | 2.20 | 2.73 |
| Soberanes | 8 | 24.2 | 1.06 | 23.08 | 25.63 |  | 2.19 | 0.32 | 1.81 | 2.66 |

­

Table S3. Summary of daily temperature profile (combined water and air) in °C during the experiment from temperature loggers inside the easternmost and westernmost cages and one mid zone site logger (PISCO; Multi-Agency Rocky Intertidal Network (MARINe) et al. 2017a, 2017b, 2018). Loggers recorded temperature every 15 min

| **Logger** | **Dates (DD/MM/YY)** | **N** | **Mean** | **Median** | **Min** | **Max** | **SD** |
| --- | --- | --- | --- | --- | --- | --- | --- |
| East cage | 26/01/18–24/07/18 | 168 | 13.83 | 13.98 | 8.63 | 17.70 | 1.64 |
| West cage | 26/01/18–01/08/18 | 188 | 13.90 | 14.01 | 8.37 | 17.20 | 1.69 |
| PISCO | 17/10/17–01/08/18 | 289 | 13.31 | 13.26 | 2.74 | 23.67 | 1.68 |

Table S4. Summary of final sizes of *Nucella* by population origin

| **Population** | **Length (mm)** | | | | |  | **Mass (g)** | | | |
| --- | --- | --- | --- | --- | --- | --- | --- | --- | --- | --- |
|  | **N** | **Mean** | **SD** | **Min** | **Max** |  | **Mean** | **SD** | **Min** | **Max** |
| Hopkins | 8 | 24.11 | 0.42 | 23.20 | 24.47 |  | 2.42 | 0.14 | 2.20 | 2.65 |
| Lompoc | 8 | 25.73 | 0.76 | 24.94 | 26.78 |  | 3.06 | 0.22 | 2.87 | 3.42 |
| Soberanes | 8 | 25.70 | 1.21 | 24.84 | 28.47 |  | 2.72 | 0.29 | 2.45 | 3.33 |

Table S5. Summary of drilled mussels by treatment

| **Treatment** | **N** | **Length (mm)** | | **Number** | | **Total Number** |
| --- | --- | --- | --- | --- | --- | --- |
|  |  | Mean | SD | Mean | SD |  |
| Control | 8 | 15.86 | 6.48 | 3.75 | 2.25 | 30 |
| Hopkins | 8 | 27.39 | 2.86 | 22.13 | 6.45 | 177 |
| Lompoc | 8 | 35.60 | 7.49 | 26.38 | 9.75 | 211 |
| Soberanes | 8 | 31.06 | 6.38 | 29.25 | 10.39 | 234 |

Table S6. Summary of remaining mussels by treatment

| **Treatment** | **N** | **Length (mm)** | | **Number** | |
| --- | --- | --- | --- | --- | --- |
|  |  | Mean | SD | Mean | SD |
| Control | 8 | 18.07 | 2.23 | 492.4 | 173.1 |
| Hopkins | 8 | 19.09 | 3.14 | 464.6 | 282.9 |
| Lompoc | 8 | 20.05 | 3.64 | 377.4 | 272.0 |
| Soberanes | 8 | 18.21 | 2.98 | 485.0 | 186.2 |

Table S7. Summary of Shannon-Wiener diversity by treatment

| **Population** | **N** | **Mean** | **SD** | **Min** | **Max** |
| --- | --- | --- | --- | --- | --- |
| Hopkins | 8 | 1.26 | 0.34 | 0.74 | 1.63 |
| Lompoc | 8 | 1.29 | 0.29 | 0.89 | 1.83 |
| Soberanes | 8 | 1.27 | 0.34 | 0.76 | 1.79 |

Table S8. Summary of permutational multivariate analysis of variance (PERMANOVA) using Bray-Curtis community distance matrix

|  | **DF** | **Sum sq** | **R^2^** | **F** | **P** |
| --- | --- | --- | --- | --- | --- |
| Block | 7 | 24.13 | 0.38 | 1.37 | 0.19 |
| *Nucella* population | 2 | 25.73 | 0.06 | 0.78 | 0.61 |
| Residual | 14 | 25.74 | 0.56 |  |  |
| Total | 23 | 1.23 | 1.00 |  |  |

Table S9. Summary of ANOVA model of *Pachygrapsus crassipes* biomass vs. *Nucella* population

|  | **DF** | **Sum sq** | **F** | **P** |
| --- | --- | --- | --- | --- |
| Block | 7 | 20.93 | 2.49 | 0.07 |
| *Nucella* population | 2 | 9.28 | 3.86 | 0.05* |
| Residuals | 14 | 16.80 |  |  |

Table S10. ANOVA table for linear regression model of *Lottia* spp. biomass vs. mean drilled mussel length

|  | Sum sq | DF | F | P |
| --- | --- | --- | --- | --- |
| Block | 13.34 | 7 | 4.14 | 0.01* |
| Mean drilled mussel length | 2.16 | 1 | 4.68 | 0.05* |
| Residuals | 6.91 | 15 |  |  |

Residual standard error: 0.6786 on 15 degrees of freedom

Multiple R-squared: 0.6598, Adjusted R-squared: 0.4783

F-statistic: 3.636 on 8 and 15 DF, p-value: 0.01502

Table S11. ANOVA table for linear regression model of *Littorina* spp. biomass vs. mean drilled mussel length

|  | Sum sq | DF | F | P |
| --- | --- | --- | --- | --- |
| Block | 1.29 | 7 | 0.87 | 0.55 |
| Mean drilled mussel length | 1.72 | 1 | 8.16 | 0.01* |
| Residuals | 3.17 | 15 |  |  |

Residual standard error: 0.4598 on 15 degrees of freedom

Multiple R-squared: 0.4886, Adjusted R-squared: 0.2159

F-statistic: 1.792 on 8 and 15 DF, p-value: 0.1573

Table S12. Structural equation model summary of *Nucella* population treatment, mean drilled mussel length, mean *Pachygrapsus crassipes* biomass, and *Littorina* spp. biomass

| Response | Predictor | Estimate | Std.Error | DF | Crit.Value | P.Value | Std.Estimate | Significance |
| --- | --- | --- | --- | --- | --- | --- | --- | --- |
| Mean drilled mussel length | trtmnt | - | - | 2 | 3.9235 | 0.0444 | - | * |
| Mean drilled mussel length | trtmnt = HOP | 27.388 | 2.0776 | 14 | 13.1825 | 0 | - | *** |
| Mean drilled mussel length | trtmnt = SOB | 31.0563 | 2.0776 | 14 | 14.9482 | 0 | - | *** |
| Mean drilled mussel length | trtmnt = LOM | 35.6029 | 2.0776 | 14 | 17.1366 | 0 | - | *** |
| Mean drilled mussel length | block | - | - | 7 | 1.0419 | 0.4463 | - |  |
| Mean drilled mussel length | block = Z | 26.2119 | 3.3927 | 14 | 7.726 | 0 | - | *** |
| Mean drilled mussel length | block = W | 28.2917 | 3.3927 | 14 | 8.339 | 0 | - | *** |
| Mean drilled mussel length | block = S | 29.9856 | 3.3927 | 14 | 8.8383 | 0 | - | *** |
| Mean drilled mussel length | block = X | 30.3889 | 3.3927 | 14 | 8.9572 | 0 | - | *** |
| Mean drilled mussel length | block = T | 30.6556 | 3.3927 | 14 | 9.0357 | 0 | - | *** |
| Mean drilled mussel length | block = U | 34.1941 | 3.3927 | 14 | 10.0787 | 0 | - | *** |
| Mean drilled mussel length | block = V | 34.5697 | 3.3927 | 14 | 10.1894 | 0 | - | *** |
| Mean drilled mussel length | block = Y | 36.495 | 3.3927 | 14 | 10.7569 | 0 | - | *** |
| *Pachygrapsus crassipes* biomass | Mean drilled mussel length | 0.0963 | 0.0443 | 13 | 2.1754 | 0.0486 | 0.4455 | * |
| *Pachygrapsus crassipes* biomass | trtmnt | - | - | 2 | 2.1785 | 0.1528 | - |  |
| *Pachygrapsus crassipes* biomass | trtmnt = SOB | 1.9403 | 0.3444 | 13 | 5.6342 | 1.00E-04 | - | *** |
| *Pachygrapsus crassipes* biomass | trtmnt = HOP | 2.6364 | 0.3862 | 13 | 6.8259 | 0 | - | *** |
| *Pachygrapsus crassipes* biomass | trtmnt = LOM | 2.9593 | 0.3923 | 13 | 7.5437 | 0 | - | *** |
| *Pachygrapsus crassipes* biomass | block | - | - | 7 | 2.5035 | 0.0727 | - |  |
| *Pachygrapsus crassipes* biomass | block = Y | 1.2838 | 0.6064 | 13 | 2.1171 | 0.0541 | - |  |
| *Pachygrapsus crassipes* biomass | block = X | 2.0253 | 0.5636 | 13 | 3.5936 | 0.0033 | - | ** |
| *Pachygrapsus crassipes* biomass | block = Z | 2.1886 | 0.6062 | 13 | 3.6101 | 0.0032 | - | ** |
| *Pachygrapsus crassipes* biomass | block = S | 2.2007 | 0.5652 | 13 | 3.8937 | 0.0018 | - | ** |
| *Pachygrapsus crassipes* biomass | block = T | 2.5065 | 0.5628 | 13 | 4.4535 | 7.00E-04 | - | *** |
| *Pachygrapsus crassipes* biomass | block = W | 2.5808 | 0.578 | 13 | 4.4648 | 6.00E-04 | - | *** |
| *Pachygrapsus crassipes* biomass | block = U | 2.9442 | 0.5759 | 13 | 5.1122 | 2.00E-04 | - | *** |
| *Pachygrapsus crassipes* biomass | block = V | 4.3663 | 0.5798 | 13 | 7.531 | 0 | - | *** |
| *Littorina* biomass | *Pachygrapsus crassipes* biomass | -0.2889 | 0.1151 | 12 | -2.5111 | 0.0274 | -0.7957 | * |
| *Littorina* biomass | Mean drilled mussel length | -0.024 | 0.0215 | 12 | -1.1207 | 0.2843 | -0.3063 |  |
| *Littorina* biomass | trtmnt | - | - | 2 | 0.3536 | 0.7092 | - |  |
| *Littorina* biomass | trtmnt = HOP | 0.9182 | 0.1609 | 12 | 5.7075 | 1.00E-04 | - | *** |
| *Littorina* biomass | trtmnt = SOB | 0.9394 | 0.1573 | 12 | 5.9719 | 1.00E-04 | - | *** |
| *Littorina* biomass | trtmnt = LOM | 1.1168 | 0.1707 | 12 | 6.5424 | 0 | - | *** |
| *Littorina* biomass | block | - | - | 7 | 2.0505 | 0.1311 | - |  |
| *Littorina* biomass | block = Y | 0.4897 | 0.2886 | 12 | 1.697 | 0.1155 | - |  |
| *Littorina* biomass | block = Z | 0.6055 | 0.2543 | 12 | 2.3814 | 0.0347 | - | * |
| *Littorina* biomass | block = X | 0.6706 | 0.2404 | 12 | 2.7891 | 0.0164 | - | * |
| *Littorina* biomass | block = S | 0.7176 | 0.2372 | 12 | 3.0251 | 0.0106 | - | * |
| *Littorina* biomass | block = T | 0.8866 | 0.2335 | 12 | 3.7971 | 0.0025 | - | ** |
| *Littorina* biomass | block = W | 1.2656 | 0.24 | 12 | 5.2746 | 2.00E-04 | - | *** |
| *Littorina* biomass | block = U | 1.458 | 0.2441 | 12 | 5.9739 | 1.00E-04 | - | *** |
| *Littorina* biomass | block = V | 1.8381 | 0.3215 | 12 | 5.7165 | 1.00E-04 | - | *** |

Fisher's C = 0 with P-value = 1 and on 0 degrees of freedom
Individual R-squared: Mean drilled mussel length 0.52; *Pachygrapsus.crassipes* 0.74; *Littorina* 0.68.

**Supplementary References**

Chan, F., J. A. Barth, C. A. Blanchette, R. H. Byrne, F. Chavez, O. Cheriton, R. A. Feely, G. Friederich, B. Gaylord, T. Gouhier, S. Hacker, T. Hill, G. E. Hofmann, M. A. McManus, B. A. Menge, K. J. Nielsen, A. Russell, E. Sanford, J. Sevadjian, and L. Washburn. 2017. Persistent spatial structuring of coastal ocean acidification in the California Current System. Scientific Reports 7:1–7.

Multi-Agency Rocky Intertidal Network (MARINe), Partnership for the Interdisciplinary Studies of Coastal Oceans (PISCO), and P. Raimondi. 2017a. MARINe/PISCO: Intertidal: site temperature data: Terrace Point (ITRPXX).
doi: ﻿10.6085/AA/ITRPXX_XXXITV2XMSR01_20170101.50.1.

Multi-Agency Rocky Intertidal Network (MARINe), Partnership for the Interdisciplinary Studies of Coastal Oceans (PISCO), and P. Raimondi. 2017b. MARINe/PISCO: Intertidal: site temperature data: Terrace Point (ITRPXX).
doi: ﻿10.6085/AA/ITRPXX_XXXITV2XMSR01_20171017.50.2.

Multi-Agency Rocky Intertidal Network (MARINe), Partnership for Interdisciplinary Studies of Coastal Oceans (PISCO), and P. Raimondi. 2018. MARINe/PISCO: Intertidal: site temperature data: Terrace Point (ITRPXX).
doi: ﻿10.6085/AA/ITRPXX_XXXITV2XMSR01_20180412.50.1.

Rivest, E. B., M. O’Brien, L. Kapsenberg, C. C. Gotschalk, C. A. Blanchette, U. Hoshijima, and G. E. Hofmann. 2016. Beyond the benchtop and the benthos: Dataset management planning and design for time series of ocean carbonate chemistry associated with Durafet®-based pH sensors. Ecological Informatics 36:209–220.
